# Study on crack resistance of self-healing microcapsules in asphalt pavement by multi-scale method

**DOI:** 10.1371/journal.pone.0300178

**Published:** 2024-03-21

**Authors:** Hongliang Zhang, Weiwen Quan, Ruixiang Wang

**Affiliations:** 1 School of Highway, Chang’an University, Xi’an, Shaanxi, China; 2 Harbin Institute of Technology, School of Transportation Science and Engineering, Harbin, China; Shandong University of Technology, CHINA

## Abstract

Self-healing microcapsules in the asphalt pavement must be kept intact under vehicle load to ensure there is enough rejuvenator in capsules when cracks appear in asphalt pavement. In this paper, the crack resistance of self-healing microcapsules in asphalt pavement was evaluated. Firstly, an expanding multi-scale analysis was conducted based on proposed mesoscopic mechanical models with the aim to determine the mechanical parameters for the following contracting multi-scale analysis. Secondly, the periodic boundary condition was introduced for the contracting multi-scale analysis and the stress field of the capsule wall was obtained. Finally, the effects of the design parameters of the microcapsule on its crack resistance in asphalt pavement were investigated. The results showed that the incorporation of microcapsules has almost no effect on the elastic constants of the asphalt mixture. The core could be simplified as an approximately incompressible solid with the elastic constants determined by the proposed mesoscopic mechanical model. With the increase of the modulus of the capsule wall, the mean maximum tensile stress of the capsule wall increased from 0.372 MPa to 0.465 MPa, while with the decrease of the relative radius of the capsule core, the mean maximum tensile stress of the capsule wall increased from 0.349 MPa to 0.461 MPa. The change in the mean maximum tensile stress of the capsule wall caused by the change of capsule diameter was within 5%. The relative radius of the capsule core and the elastic modulus of capsule wall were two key parameters in capsule design. Besides, the microcapsules with the wall made of resin would not crack under the vehicle load before microcracks occurred in asphalt pavement.

## 1. Introduction

In order to alleviate the cracks caused by vehicle and temperature variation, self-healing asphalt pavement have been proposed. The self-healing ability of asphalt pavement itself is small, so the serious cracks caused by heavy vehicle loads, low temperature or serious aging can not be self-healed. There are two commonly accepted methods to promote the healing of the cracks in asphalt pavements. One is the use of polymer modifiers or external assistance for compositing self-healing asphalt materials; the other is to add materials with higher electrical conductivity, such as metal fibers, into asphalt mixtures to increase the inner temperature of mixtures when energized [1–4]. Microcapsule is one of the most commonly used auxiliary agents.

Recently, studies have concentrated on the preparation, mechanical properties, and the self-healing efficiency of microcapsules. Some scholars prepared microcapsules from a rejuvenator and a resin through in situ polymerization and investigated the effects of the capsule size, the thickness of the capsule wall and the preparation process on the self-healing efficiency of microcapsules [2–4]. Based on micro-manipulation techniques, Wang and Hu systematically investigated the mechanical strength parameters of urea-formaldehyde microcapsules and melamine-formaldehyde (MF) encapsulated dicyclopentadiene (DCPD) microcapsules, respectively [5–7]. Su et al. obtained the relationship between the mechanical properties and shell thickness of the microcapsules, as well as the elastic modulus of the shell by delineating the load-displacement characteristics through nano-indentation assays [8,9].

Mechanical analysis on microcapsules in composite materials were carried out by a few scholars. Zhao [10] investigated the crack propagation in resin matrix containing microcapsules, and discussed the relationship between the capsule rupture and model parameters such as the elastic modulus of the capsule wall, the strength of wall material and the offset distance of the crack. However, the microcapsule was assumed to be hollow during simulation and the effect of capsule core on mechanical responses of capsule wall was ignored. Meanwhile, the load applied on the model was determined according to the laboratory load, which is quite different from the actual vehicle load applied on asphalt pavements. Quayum et al. [11] predicted the equivalent elastic modulus of self-healing concrete based on inclusion theory and representative volume element (RVE). Based on cohesive elements, Mauludin et al. [12] found that as capsules volume ratio or the capsules core-shell thickness ratio increased, the fracture probability of the capsules increased. Papaioannou et al. [13] applied Monte Carlo simulation to investigate the potential of capsules to heal cracks of different widths and found that 10% vol. of capsules with a diameter of 3±0.3 mm could provide an adequate amount of healing agent while simultaneously contributing to load regain of the cementitious matrix under flexural stress. Gilabert et al. [14] adopted extended finite element method (XFEM) and cohesive surfaces (CS) technique to predict crack propagation in a three-point bending concrete beam containing microcapsules. However, indoor experiment load rather than the actual load in structure were adopted during simulation in the above studies.

In order to ensure that microcapsules only crack when they encounter cracks in the road, microcapsules should meet the requirements of crack resistance. In other words, microcapsules should be able to withstand the vehicle load in the early service stage of the asphalt pavement. Since the diameter of the microcapsule is between 50 and 200 μm and the stress field of the microcapsule is complicated, it is difficult to obtain the stress distribution of the microcapsule through experiments. Therefore, the numerical analysis method is the only feasible way to achieve this goal. However, due to the large difference in size between microcapsules and pavement structure, a huge occupation of the computational source will occur if traditional macroscopic numerical analysis method is conducted. To improve the efficiency of numerical analysis on the large model containing microstructures, multi-scaling analysis methods were introduced by scholars. Multi-scaling methods aim at realizing the transfer of mechanical parameters and stresses between different scales of models, where expanding and contracting multi-scale are included. The former can obtain the equivalent mechanical parameters of the macro models by the homogenization process on the mesoscopic or micro model, while the latter can get the stress exerted on the mesoscopic or micro model [15–17]. For the analysis on the microcapsules in asphalt pavement, because of the technical difficulties in the realization of the contracting multi-scale analysis by commercial numerical simulation software, only a few studies were carried out. A multi-scaling model of self-healing asphalt pavements was established by Zhu [18]. In the first step of his study, a macro model containing coarse aggregates and mortars was established by the discrete element method (DEM), and the average contact pressure and tensile force exerted on mortars were obtained through scalar operations. In the second step, a mesoscopic model included asphalt mortars and microcapsules was established by DEM, and the contact pressure and tensile stress exerted on a single capsule were obtained. Finally, this contact pressure was applied to the microcapsule through virtual parallel plate test by finite element software. Among all of the steps in his study, the vector property of the force was neglected. Moreover, the simplification of the capsule core as an incompressible solid and the boundary condition of mesoscopic models were not rational.

Recently, multi-scaling methods have developed quickly, and among them, the inclusion theory can be applied to predict the equivalent mechanical parameters of materials, and the periodic boundary conditions can achieve the stress transfer between macroscopic and mesoscopic models. Based on the two-phase micromechanical model (MTPMM), Peng et al [19] predicted the dynamic modulus of asphalt mixture. Sun et al [20] predicted the viscoelastic behavior of asphalt concrete from mesomechanical viewpoint based on two two-dimensional calculation methods. Xia et al. and Sharma [21,22] adopted the nodal displacement method to apply periodic boundary conditions to the mesoscopic representative volume element (RVE) and predicted the equivalent elastic constants of the fiber-filled composite. Yuan and Fish [23] applied the periodic boundary conditions on the mesoscopic RVE by thermal strain method and realized the stress bridging in the multi-scaling models, as a result obtained the stress field of the mesoscopic model.

The objective of this study is to adopt the multi-scale methods to investigate the crack resistance of self-healing microcapsules in asphalt pavement. Firstly, expanding multi-scale analysis was conducted, several meso-mechanical models were proposed to determine the optimum mechanical parameters for the following contracting multi-scale analysis. Then, a contracting multi-scale method named periodic boundary condition was introduced and used in ABAQUS, and its rationality for the contracting multi-scale was validated. After that, with the help of the submodel method and the periodic boundary condition, the contracting multi-scale analysis was conducted for obtaining the stress field of the capsule wall. Finally, effects of the design parameters of the capsule on the stress field of the capsule wall were investigated, and the crack resistance of microcapsules in asphalt pavement was furtherly validated.

## 2. Research methodology

### 2.1 Mesoscopic model of microcapsules

Hollow sphere model and Hanshin model were adopted to predict the equivalent elastic modulus of the capsule and determine the optimum elastic constants of the capsule core by comparisons. In the hollow sphere model, the capsule core was regarded as a liquid, while in the Hanshin model it was regarded as an approximately incompressible solid. To facilitate the following derivation, the conversion relationships of elastic constants of isotropic materials were given as follows:

$$K=\frac{E}{3(1-2\nu )}$$ (1)

$$G=\frac{E}{2(1+\nu )}$$ (2)

where *K*, *E*, *G* and *ν* represented the bulk modulus, the elastic modulus, the shear modulus and the Poisson’s ratio, respectively.

#### 2.1.1 Hollow sphere model

The equivalent bulk and shear moduli of the hollow sphere model could be obtained via Eqs (3) and (4) [24].

$$K_{hom}^{S}=\frac{4K_{m}G_{m}(1-c)}{4G_{m}+3cK_{m}}$$ (3)

$$\frac{G_{hom}^{s}}{G_{m}}=\frac{-B+\sqrt{B^{2}-AC}}{A}$$ (4)

where $K_{hom}^{S}$ and $G_{hom}^{S}$ were equivalent bulk modulus and shear modulus predicted by hollow sphere model, respectively; *c* was the inclusion ratio, the subscripts of *hom* and *m* represented matrix and homogeneity, respectively; *A*, *B*, and *C* were the parameters related to the mechanical and geometry properties of the matrix and inclusion, whose details could be found in the reference[24].

Based on hollow sphere model, a prediction process of the equivalent elastic constants of the microcapsule was as follows:

Firstly, the liquid capsule core was replaced by a hollow sphere model consisting of a solid capsule wall and a void. Under this replacement, the bulk modulus of liquid was regarded as the equivalent bulk modulus, and the modulus of the capsule wall was regarded as the matrix modulus. Therefore, the converted void volume ratio named *c*_*w*_ could be obtained via Eq (5).

$$c_{w}=\frac{4G_{w}(K_{w}-K_{c})}{K_{w}(3K_{c}+4K_{w})}$$ (5)

where *K*_*w*_, *G*_*w*_ and *K*_*c*_ were the bulk modulus of the capsule wall, the shear modulus of the capsule wall and the the bulk modulus of the capsule core, respectively.

Secondly, based on the *c*_*w*_ and the initial core volume ratio named *c*_*o*_, the modified core ratio named *c*_*m*_ could be obtained via Eq (6). Then, substituting elastic modulus of the capsule wall and *c*_*m*_ into Eq (3), the equivalent bulk modulus of the capsule could be obtained.


$$c_{m}=c_{o}c_{w}$$ (6)

Thirdly, assuming that the shear resistance of liquid was small enough to be ignored. In other words, its shear modulus was zero. When the shear modulus of capsule core, the shear modulus of capsule wall and the initial core volume ratio were known, the equivalent shear modulus of capsule could be obtained from Eq (4).

Finally, based on Eqs (1) and (2), the equivalent elastic modulus and Poisson’s ratio of the capsule could be obtained.

#### 2.1.2 Hanshin model

In Hanshin model, the capsule wall and the capsule core should be regarded as the matrix and the inclusion, respectively. If the elastic constants of the capsule core, the elastic constants of the capsule wall, and the volume ratio of the capsule core were known, the equivalent elastic constants of the capsule could be determined from Eqs (4) and (7). After that, based on Eqs (1) and (2), the equivalent elastic modulus and Poisson’s ratio of the capsule could be obtained.

$$K_{hom}^{H}=K_{m}+\frac{c(K_{i}-K_{m})(3K_{m}+4G_{m})}{3K_{m}+4G_{m}+3(1-c)(K_{i}-K_{m})}$$ (7)

where $K_{hom}^{H}$ represented the equivalent bulk modulus predicted by the Hanshin model. The meaning of other parameters could be found below Eq (4).

#### 2.1.3 Equivalent elastic constants of asphalt mixture with capsules

Based on the hollow sphere model, the microcapsule can be homogenized into a uniform sphere with equivalent elastic constants. In this section, the microcapsule and asphalt mixture were regarded as the inclusion and the matrix, respectively. Therefore, the equivalent elastic constants of asphalt mixture with capsules could be obtained from Eqs (7) and (8).


$$G_{hom}=G_{i}+\frac{5cG_{m}(G_{i}-G_{m})(3k_{i}+4G_{i})}{5G_{i}(3k_{i}+4G_{i})+6(1-c)(G_{i}-G_{m})(k_{m}+2G_{m})}$$ (8)

### 2.2 Method for contracting multi-scale analysis

**Fig 1:**
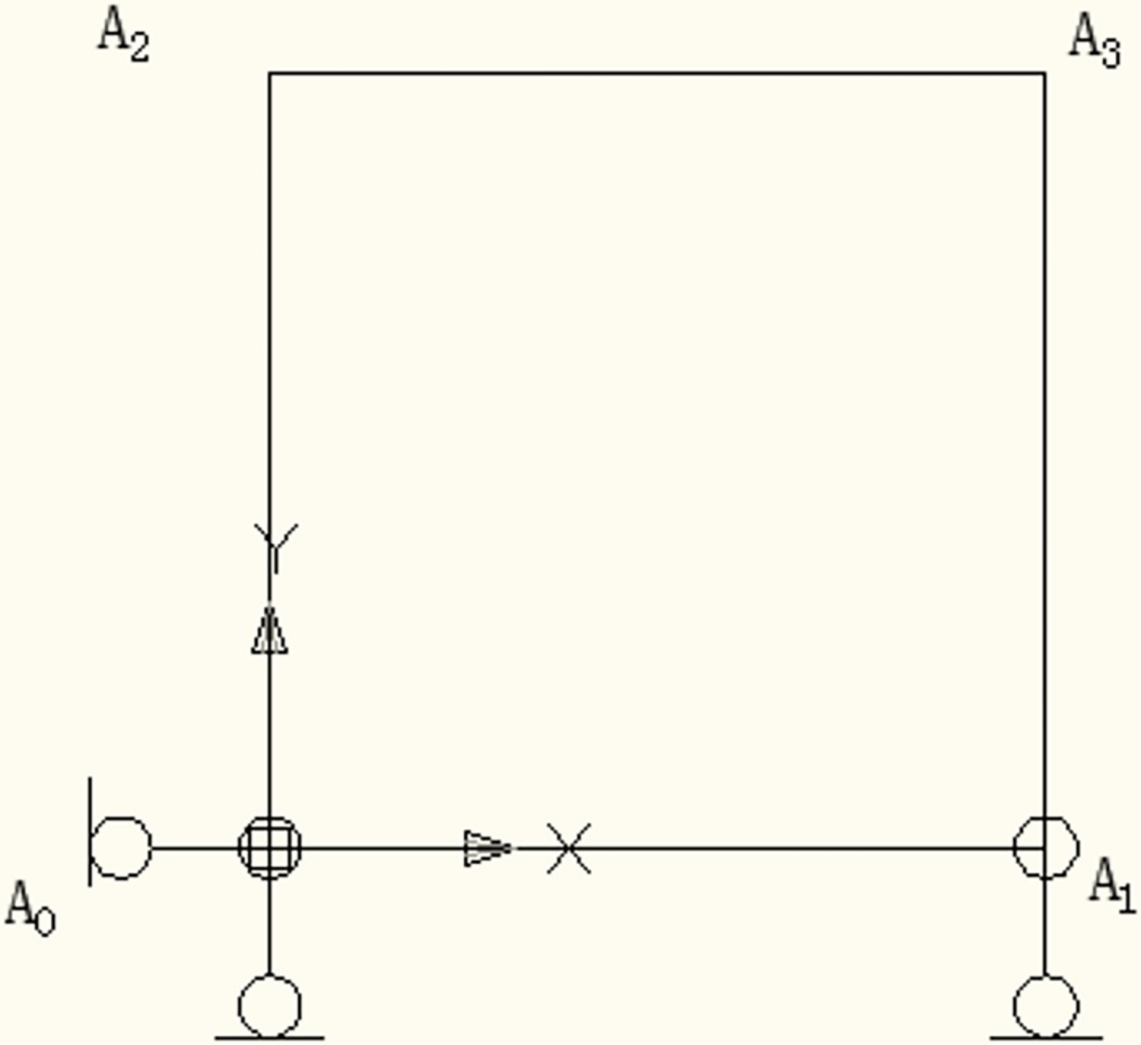
Two-dimensional RVE.

The periodic boundary condition was adopted for the contracting multi-scaling analysis. Though this method has been used in the analysis of composite materials, it has not been utilized in the analysis of asphalt pavement. In this paper, the realization process of periodic boundary conditions in ABAQUS is as follows:

As shown in Fig 1, a two-dimensional rectangle RVE had two pairs of opposite boundary faces.

Assuming that the RVE was subject to a periodic displacement field, the displacement of the nodes on the RVE’s faces should satisfy Eqs (9) and (10).

$$u_{i}^{j+}={\bar{\varepsilon }}_{ik}x_{k}^{j+}+u_{i}^{*}$$ (9)

$$u_{i}^{j+}={\bar{\varepsilon }}_{ik}x_{k}^{j+}+u_{i}^{*}$$ (10)

where ${\bar{\varepsilon }}_{ik}$ was the average strain tensor which could be obtained from the integration point of the macro model, *x*_*k*_ was the coordination of a given node, $u_{i}^{*}$ was the correction of the periodic displacement, *j*+ and *j*− represented the normal directions of the RVE’s faces.

Subtracting Eq (9) from Eq (10) yielded:

$$u_{i}^{j+}-u_{i}^{j-}={\bar{\varepsilon }}_{ik}(x_{k}^{j+}-x_{k}^{j-})={\bar{\varepsilon }}_{ik}\Delta x_{k}^{j}$$ (11)

In order to prevent the rigid body displacement of the two-dimensional RVE, displacements of some nodes were fixed according to Fig 1. The displacement of *A*_0_ in the *x* and *y* directions and that of *A*_1_ in the y direction were set to be zero. Based on the above constraints, all the shear strains were applied on the face with the *y*-axis as the external normal line, and the displacements of the main node (*A*_1_ and *A*_2_) were adopted to replace the right side of the above-mentioned displacement constraint equations. Eventually, the displacement equations of nodes (except four nodes on the corner) could be transformed into:

$$u_{1}^{1+}-u_{1}^{1-}={\bar{\varepsilon }}_{11}\Delta x_{1}^{1}=u_{1}^{A_{1}}$$ (12)

$$u_{2}^{1+}-u_{2}^{1-}=u_{2}^{A_{1}}=0$$ (13)

$$u_{1}^{2+}-u_{1}^{2-}=2{\bar{\varepsilon }}_{12}\Delta x_{2}^{2}=u_{1}^{A_{2}}$$ (14)

$$u_{2}^{2+}-u_{2}^{2-}={\bar{\varepsilon }}_{22}\Delta x_{2}^{2}=u_{2}^{A_{2}}$$ (15)

where $u_{1}^{A_{1}}$ was the displacement of node *A*_1_ in *x* direction, $u_{2}^{A_{2}}$ was the displacement of *A*_*2*_ in *y* direction.

As for the nodes on the corner of the RVE, since the displacements of *A*_0_, *A*_1_ and *A*_2_ had been given, the displacement constraint equations of the remaining corner nodes were:

$$u_{1}^{A_{3}}-u_{1}^{A_{0}}={\bar{\varepsilon }}_{11}\Delta x_{1}^{1}+2{\bar{\varepsilon }}_{21}\Delta x_{1}^{1}=u_{1}^{A1}+u_{1}^{A_{2}}$$ (16)

$$u_{2}^{A_{3}}-u_{2}^{A_{0}}={\bar{\varepsilon }}_{22}\Delta x_{2}^{2}=u_{2}^{A_{2}}$$ (17)

Based on the above equations, it could be known that the node density on the opposite faces of the RVE should be the same, which meant that the periodic mesh was needed for RVE. Therefore, a meshing software named Hypermesh was adopted to obtain the desirable mesh. Moreover, a Python script was created to recognize the position of the nodes and apply the constraint Eqs (12)–(17).

### 2.3 Algorithm for the generation of mesoscopic geometric model

**Fig 2:**
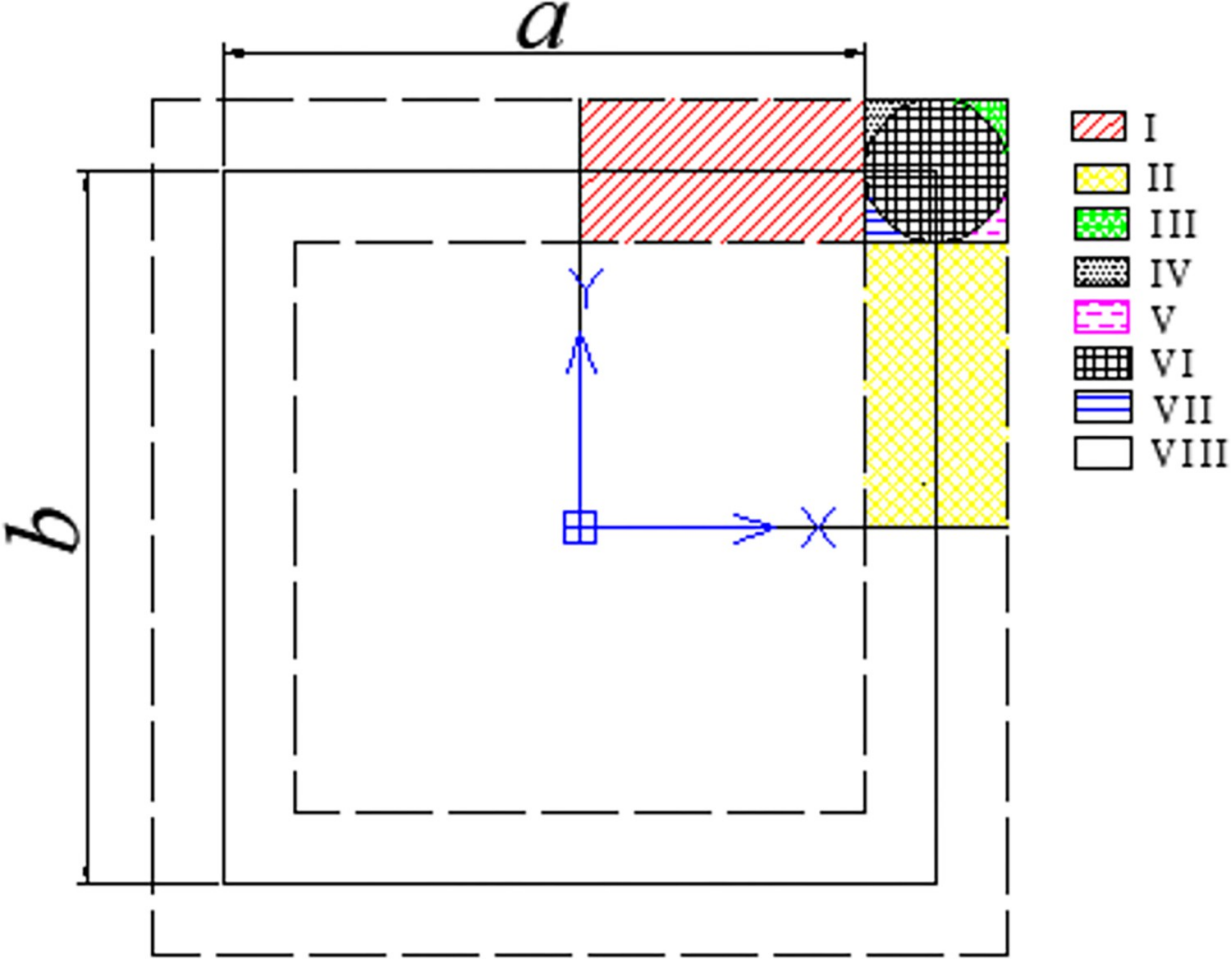
Algorithm for generation of the periodic geometric model.

The mesoscopic model consisted of asphalt mixture and some microcapsules. To establish the mesoscopic model, an algorithm to create a two-dimensional periodic geometric model was put forward. Fig 2 was adopted to illustrate the algorithm, where *a* and *b* represented the width and length of the model, respectively. The main steps of the algorithm were listed as follows:

Firstly, original particles were generated randomly within the outer dotted line, and then were transformed to the first quadrant via Eqs (18) and (19). The transformed coordinate was used to determine the mapping relationship of the original particles as shown in Table 1.

$$x_{t}=|x_{0}|$$ (18)

$$y_{t}=|y_{0}|$$ (19)

where the subscript of *t* and *o* represented transformed particles and the original particle, respectively.

**Table 1:** Mapping relationships of transformed particles.

Region	Whether to retain original particle	Number of mapping particle	Mapping relationship
I	Yes	1	Longitudinal symmetry
II	Yes	1	Bilateral symmetry
III	No	3	Longitudinal, bilateral and diagonal symmetry
IV	Yes	2	Longitudinal and diagonal symmetry
V	Yes	2	Bilateral and diagonal symmetry
VI	Yes	3	Longitudinal, bilateral and diagonal symmetry
VII	Yes	2	Longitudinal and bilateral symmetry
VIII	Yes	None	None

In the above mapping relationship, the mapping particles originating from the bilateral symmetry had the same coordination in y-direction with original particle, while the coordination relationships between original and mapping particles in x-direction were

$$x_{m}=x_{0}-a,x_{0}>0$$ (20)

$$x_{m}=x_{0}+a,x_{0}<0$$ (21)

where the subscript of *m* and *o* represented mapping particles and the original particle, respectively.

The mapping particles originating from the longitudinal symmetry had the same coordination in x-direction with the original particle, while the coordination relationships between the original and mapping particles in y-direction were as follows:

$$y_{m}=y_{0}-b,y_{0}>0$$ (22)

$$y_{m}=y_{0}+b,y_{0}<0$$ (23)

With regards to the mapping particle originating from diagonal symmetry, the coordination relationship between original and mapping particles should meet Eqs (23)–(26).

After the generation of the original and mapping particles, an overlapping judgement would be adopted to ensure all the existing particles were separate. The coordinates of all the separate particles would be recorded to generate the geometric model.

## 3. Results and analysis

### 3.1 Determination of the equivalent elastic constants of the mesoscopic model

#### 3.1.1 Equivalent elastic constants for microcapsule of mesoscopic model

It could be seen from the prediction process of the hollow sphere and the Hanshin models that, the differences between these two models lied in the simplification of the capsule core. When Hanshin model was adopted, the shear modulus of the capsule core could not be neglected. One would ask, how to determine the optimum shear modulus of the capsule core. Some useful information could be found in the cell mechanic, where it was proven that the approximate incompressible solids could be used to simulate the mechanical behavior of liquids in a closed cavity [18]. But how to determine the shear constants of the capsule core for different types of liquids was still unclear.

In this paper, the shear modulus of the capsule core was obtained from its bulk modulus. Firstly, a Poisson’s ratio of the capsule core was selected. After that, based on the known bulk modulus, the shear modulus of the capsule core could be derived from Eq (2). To validate this process, the equivalent elastic constants of the capsule got from the hollow sphere model and the Hanshin model were compared.

Regardless of whether the core was simplified to be an approximately incompressible solid or a liquid, its bulk modulus was considered to be 2.25 GPa which was the bulk modulus of the pure water in 20°C [25]. It was known from the elastic mechanic that the Poisson’s ratio of the approximately incompressible solid should be close to 0.5. However, if the Poisson’s ratio was 0.5, it was known from Eqs (1) and (2) that the elastic constants could not be converted. Therefore, for Hanshin model, Poisson’s ratios of 0.490 and 0.4999 were adopted, and the corresponding elastic modului were 0.135 and 0.0013, respectively. Besides the elastic constants of the capsule core, other independent variables involved in determining the equivalent elastic constants of the capsule were the volume ratio of the capsule core and the elastic constants of the capsule wall. In this analysis, their values were selected according to references [6–8,10,26,27]. To reduce the number of cases in analysis, two kinds of the volume ratio of capsule core were selected and the elastic constants of the capsule wall were changed in a range, as shown in Table 2.

**Table 2:** Parameters of the capsule under different cases.

Case	Volume ratio of capsule core	Poisson’s ratio of capsule core	Elastic modulus of capsule core (GPa)	Elastic modulus of capsule wall (GPa)	Poisson’s ratio of capsule wall
1	0.125	0.49	0.135	2.5–4.0	0.33–0.42
2	0.125	0.4999	0.00135		
3	0.729	0.49	0.135		
4	0.729	0.4999	0.00135		

**Fig 3:**
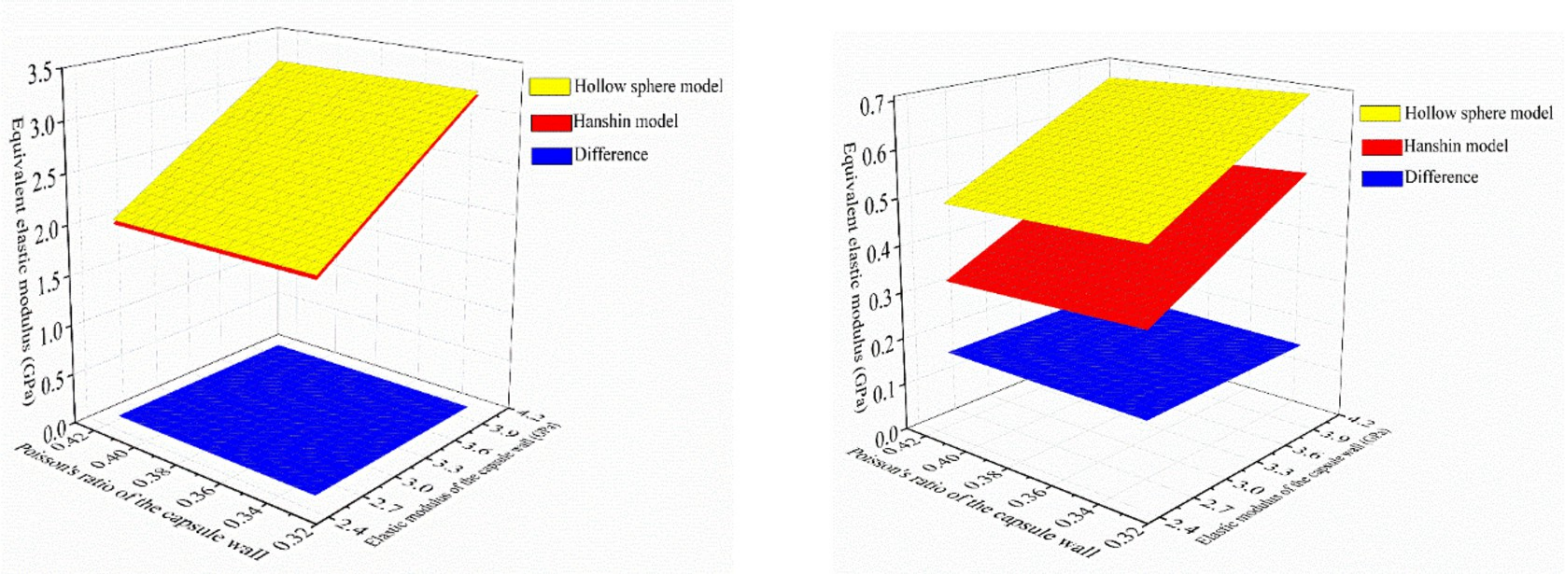
Equivalent elastic modulus of the capsule under case 1 and case 3.

**Fig 4:**
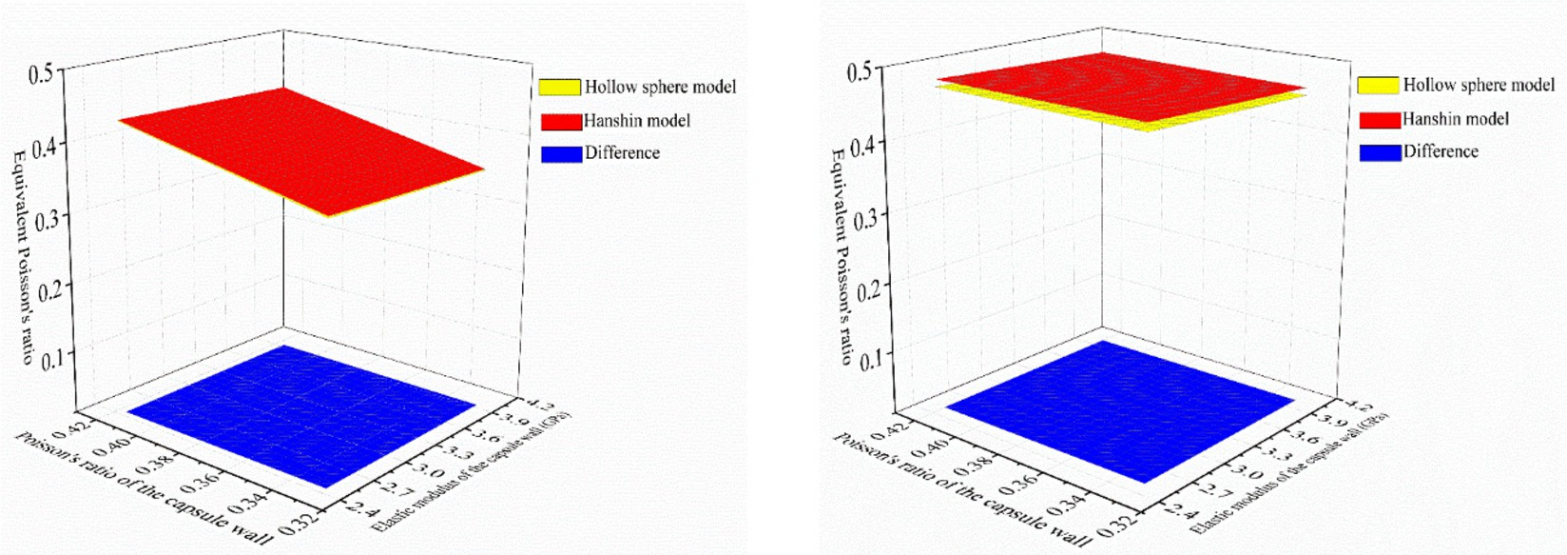
Equivalent Poisson’s ratio of the capsule under case 1 and case 3.

Because the core of the self-healing microcapsule is usually in the form of liquid, the prediction results of the hollow sphere model was regarded as the true values in the following discussion. Prediction results of the two models and differences under different cases in Table 2 were calculated. For conciseness, results of case 1 and case 3 were plotted in Figs 3 and 4. It could be seen that with an increasing elastic modulus of the capsule wall and a decreasing volume ratio of the capsule core, the equivalent elastic modulus of the microcapsules increased remarkably. The equivalent Poisson’s ratio of the capsule showed an increases trend with the increase of the volume ratio of the capsule core.

Subsequently, T-tests were conducted on the prediction results of these two models to test whether there were significant differences between each model under different cases in Table 2. It could be known that the equivalent elastic modulus, at a 5% significance level, presented p-values of 0.107, 0.987, 0 and 0.671 for cases 1 to 4, respectively. While for the equivalent Poisson’s ratio, at a 5% significance level, presented p-values of 0.227, 0.990, 0, 0.631 for cases 1 to 4, respectively. Such results may allow concluding that the difference of prediction results between these two models could be ignored under cases in Table 2 except cases 3. In other words, when the volume ratio of the capsule core is 0.729 and the elastic constants of the capsule core were determined as those in case 3, the difference of the prediction results of the two models are significant.

To better clarity the difference between different models, the maximum differences of equivalent elastic constants between different models were extracted, as shown in Table 3. It could be seen from the comparison between cases 1 and 2 or between cases 3 and 4 that when the volume ratio of the capsule core was the same, the Hanshin model would result in less difference as the Poisson’s ratio was closer to 0.5. Besides, from the comparison between cases 1 and 3 or between cases 2 and 4 that when the Poisson’s ratio of the capsule core was the same, the Hanshin model would result in more difference with the increase of the volume ratio of the capsule core. However, combined with the results from T-tests and Table 3, one could conclude that when the Poisson’s ration of the capsule core was 0.4999, the difference produced by assuming the capsule core was approximately incompressible could be neglected. In summary, if the core was simplified into an approximately incompressible solid in numeric simulation, when the bulk modulus of the capsule core was 2.25 GPa, the elastic modulus of the core was recommended to be 0.0013 GPa, and its Poisson’s ratio was 0.4999.

**Table 3:** Maximum differences of equivalent elastic constants of capsule predicted by two types of models.

Case	Poisson’s ratio (10^−3^)	Elastic modulus (MPa)
1	2.80	41.4
2	0.01	0.40
3	11.8	168.4
4	0.10	1.80

#### 3.1.2 Equivalent elastic constants for asphalt mixture with capsules of mesoscopic model

From Eqs (7) and (8), it could be concluded that when the elastic constants of the mixture were given, the equivalent elastic constants of the self-healing asphalt mixture were affected by the amount and equivalent elastic constants of the microcapsule. Because the volume ratio of microcapsules in the asphalt mixture was generally less than 0.8% [18], the following studies were conducted based on it. Besides, other required parameters were listed in Table 4. It could be obtained from the previous study that these parameters would result in the maximum or minimum equivalent elastic modulus of the capsule.

**Table 4:** Parameters of the capsule under special conditions.

Case	The volume ratio of capsule core	Poisson’s ratio of capsule wall	Elastic modulus of capsule wall (GPa)	Bulk modulus of capsule core (GPa)
1	0.125	0.33	4.0	2.25
2	0.729	0.33	2.5	

With the hollow sphere model and Mori-Tanaka model, the equivalent elastic constants of the self-healing asphalt mixture were calculated, and the results were listed in Table 5 (values in brackets represented the elastic constants of AC-13 asphalt mixture without microcapsules at 20°C). It could be seen from Table 5 that when the elastic modului of the capsule wall were 2.5 GPa and 4.0GPa, the incorporation of the microcapsules could slightly reduce and increase the equivalent elastic modulus of the self-healing mixture, respectively. But generally, the effect of the incorporating of the microcapsules on the elastic constants of asphalt mixture was small enough to be ignored.

**Table 5:** Elastic constants of self-healing asphalt mixture at 20°C.

Elastic constants	case	Mixture with capsules (without capsules)
Equivalent elastic modulus (MPa)	1	1828 (1820)
	2	1805 (1820)
Equivalent Poisson’s ratio	1	0.311 (0.311)
	2	0.313 (0.311)

### 3.2 Rationality of the periodic boundary condition in contracting multi-scaling analysis

A full-scale model (only a direction numeric simulation (DNS) model was included, which was a periodic array of a single mesoscopic RVE) and a two-scale model (including a macroscopic model and a mesoscopic RVE) based on the periodic boundary condition were established. By comparing the macroscopic displacement field and the mesoscopic stress field of the two types of models, the accuracy and advantage of applying the periodic boundary condition in multi-scale calculations would be verified. However, in pavement engineering, a lot of mesoscopic RVEs are needed in the construction of the DNS model due to the large size of pavement structure, which makes it hard to verify the rationality of the periodic boundary condition adopted in the contracting multi-scale analysis. Therefore, a SiC/Ti cantilever beam was adopted in this section.

#### 3.2.1 Prediction of the equivalent elastic constants of SiC/Ti cantilever beam

The parameters of matrix and inclusion adopted in this study were shown in Table 6 [23].

**Table 6:** Material properties for fibrous mesoscopic RVE.

Material	Elastic modulus (GPa)	Poisson’s ratio	Volume ratio
SiC fiber	379.2	0.21	0.267
Ti matrix	68.9	0.33	0.733

The analysis of the cantilever beam was simplified to a plane strain problem. The length and width of the cantilever beam were 112 mm and 44 mm, respectively. The mesoscopic RVEs were square, and their length was 4 mm. On the macroscopic scale, the SiC/Ti was regarded as a homogeneous orthotropic material. If the average stress and strain of the mesoscopic RVE were adopted to represent the responses at the integral point of the macro model, the physical equation could be expressed by Eq (24). In addition, Xia et al. [21] had put forward the relationship between the average stress component of the mesoscopic RVE and the reaction force of the main node, which was expressed as follows:

$$\left[\begin{array}{ccc} C_{11} & C_{12} & 0\\ C_{12} & C_{22} & 0\\ 0 & 0 & C_{44} \end{array}][\begin{array}{c} {\bar{\varepsilon }}_{x}\\ {\bar{\varepsilon }}_{y}\\ {\bar{\gamma }}_{xy} \end{array}]=[\begin{array}{c} {\bar{\sigma }}_{x}\\ {\bar{\sigma }}_{y}\\ {\bar{\tau }}_{xy} \end{array}\right]$$ (24)

$${\bar{\sigma }}_{ij}=\frac{{\left(P_{i}\right)}_{j}}{S_{j}}(i,j=1,2)$$ (25)

where (*P*_*i*_)_*j*_ represented the reaction force of the main node in *i* direction and this node is on the face in *j* direction, *S*_*j*_ is the area of the principal face in *j* direction.

Therefore, two sets of independent strain vectors were applied to obtain the four stiffness coefficients in Eq (25). Eventually, the four in-plane stiffness coefficients (MPa) of the SiC/Ti composite were shown in the Formula (26).


$$\left[\begin{array}{ccc} 136145 & 58537.9 & 0\\ 58537.9 & 136160 & 0\\ 0 & 0 & 35102 \end{array}\right]$$ (26)

#### 3.2.2 Mechanical responses of the DNS and two-scale models

**Fig 5:**
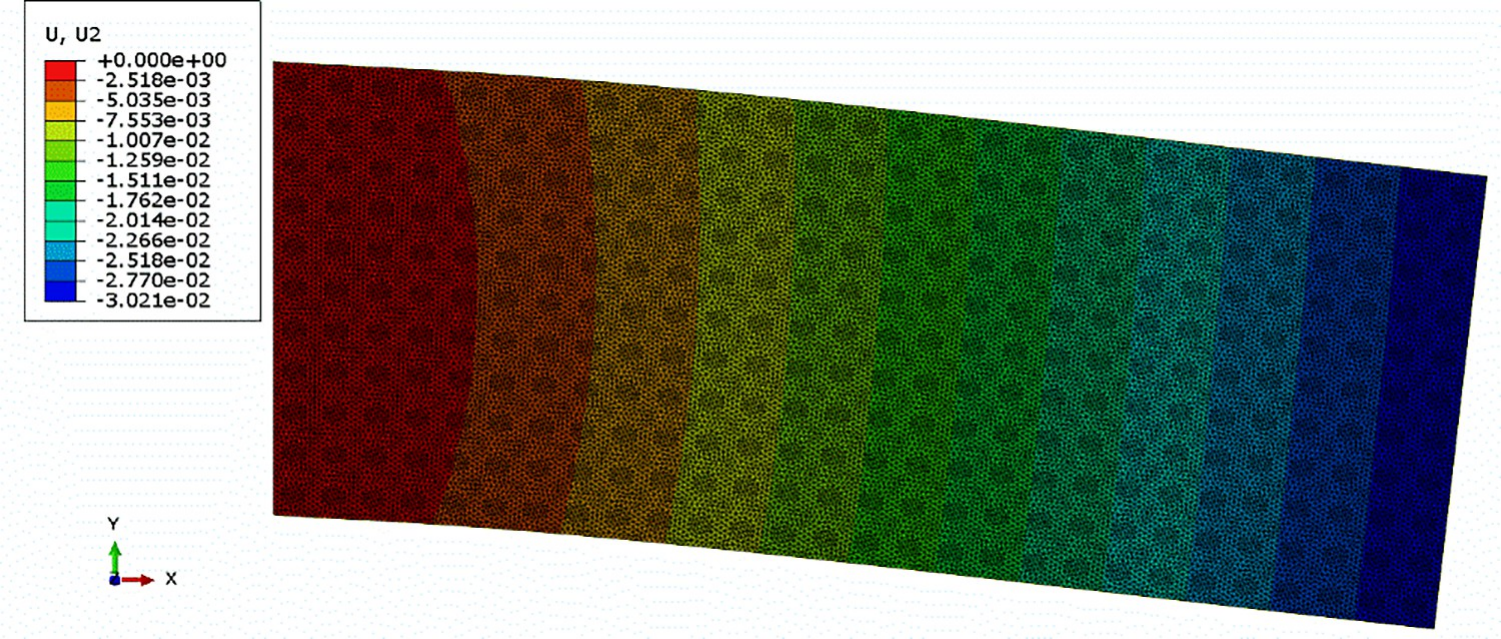
Vertical displacement of the DNS model.

The material properties were assigned to DNS model according to Table 6. The boundary conditions were set as follows: one end of the beam was fixed, and a uniform load (1 MPa) was applied on its top face in y direction. The element type of the model was CPE3, and the vertical displacement field of the DNS model was shown in Fig 5.

**Fig 6:**
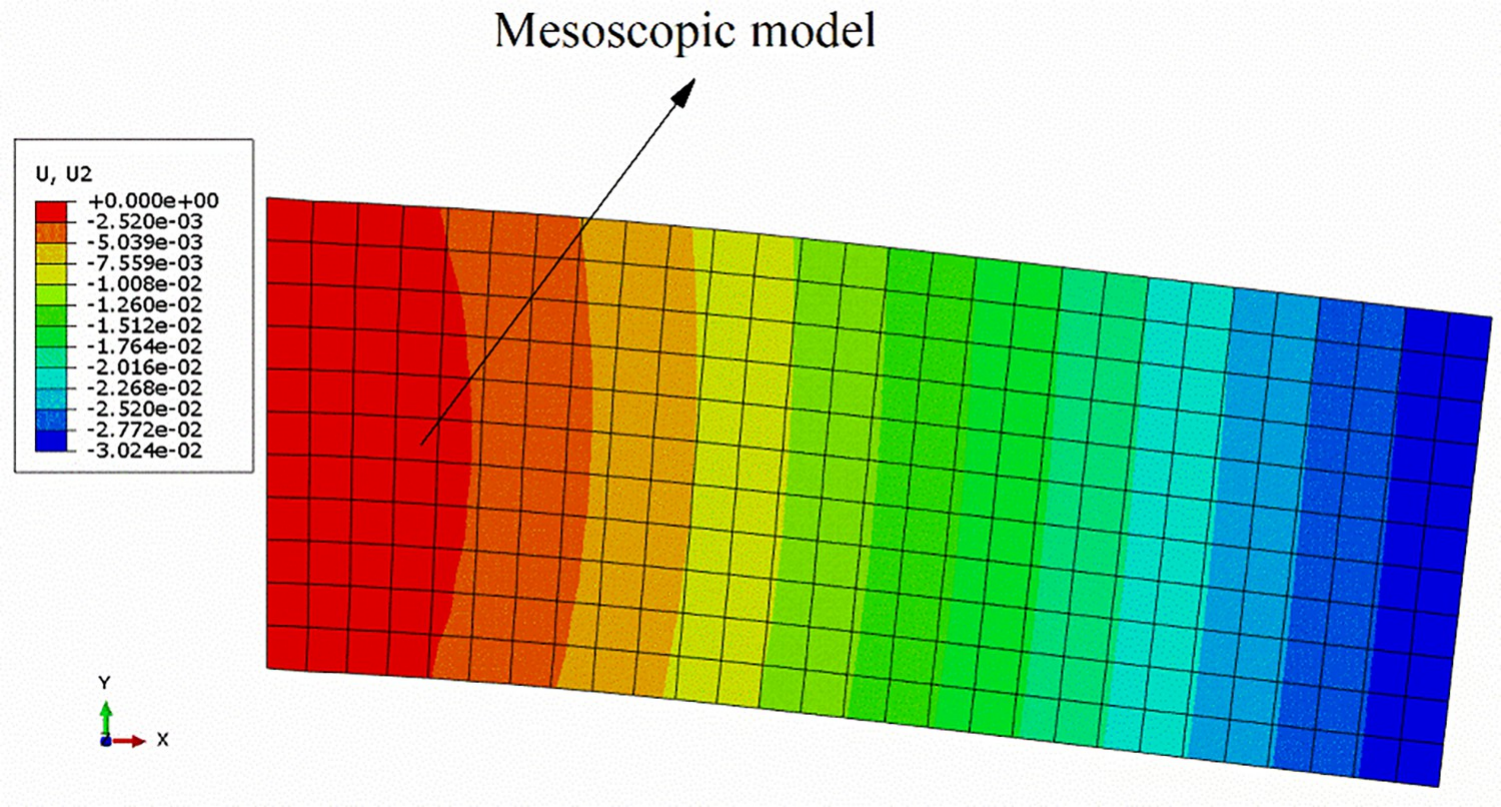
Vertical displacement field of the macro model.

The material properties of the macro model were given according to Eq (26). Its boundary conditions were consistent with the DNS model. Its element type was CPE4R, and its element size was the same as that of a single mesoscopic RVE. Finally, the vertical displacement field of the two-scale macro model was shown in Fig 6.

**Fig 7:**
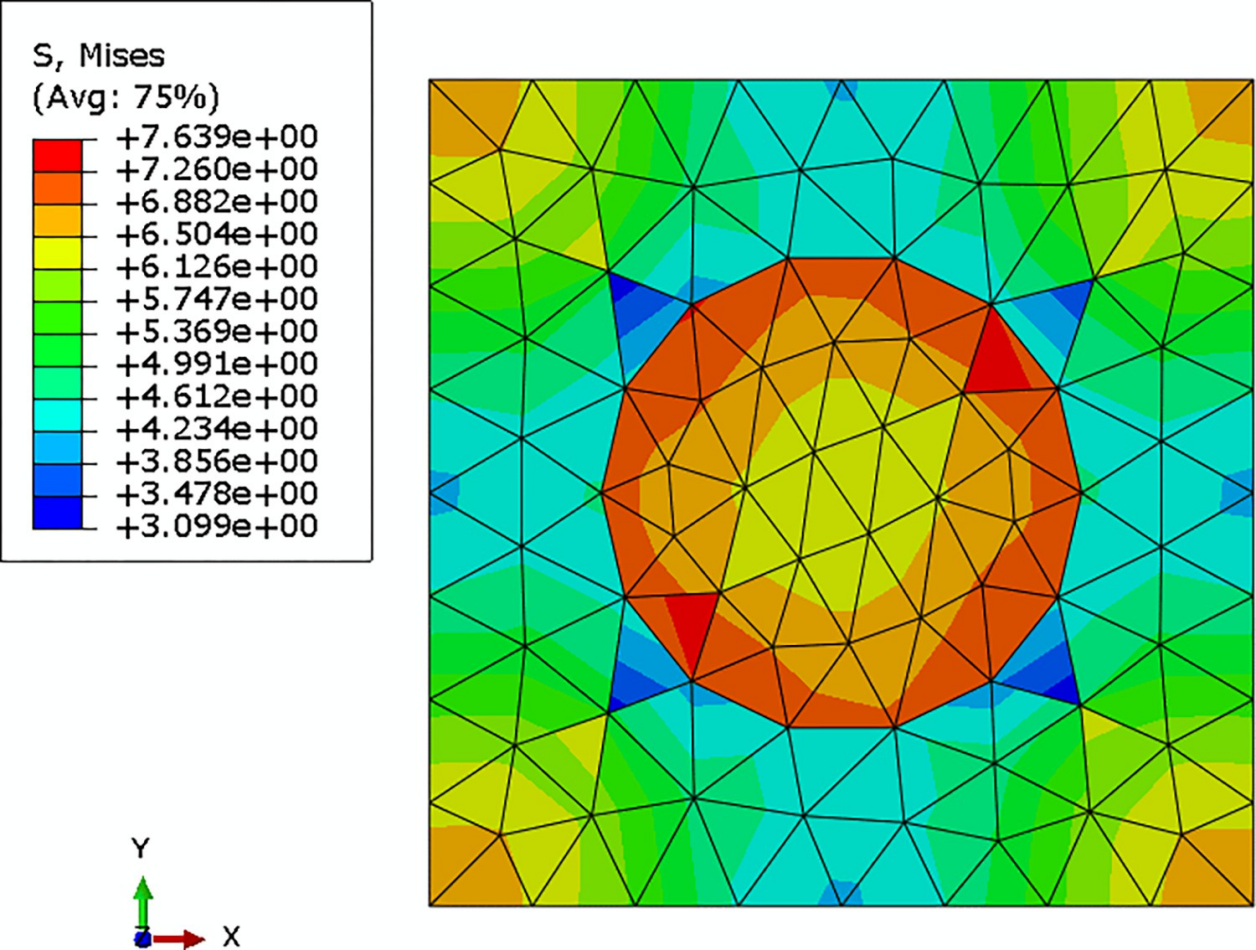
Mises stress field of the mesoscopic RVE.

The average strain component of an integration point of the macro model (as shown in Fig 6) was applied to the mesoscopic RVE by applying the periodic boundary condition, and the Mises stress field of the RVE was shown in Fig 7.

#### 3.2.3 Comparison of the results from DNS and two-scale models

**Fig 8:**
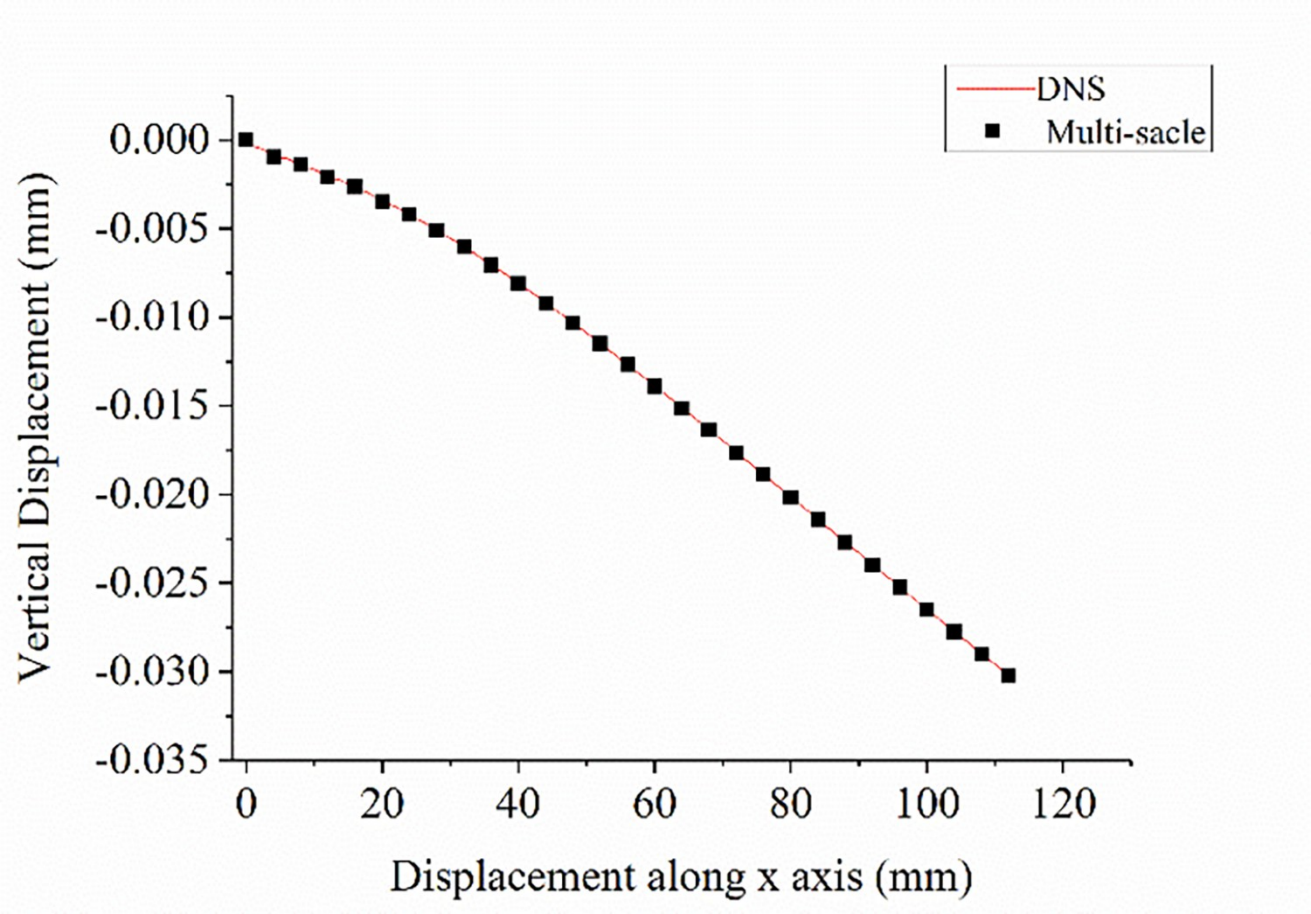
Deflection curves of the cantilever beam.

According to Figs 5 and 6, the deflection curves of the beam from the two models were plotted, as shown in Fig 8. It could be seen that the two defection curves were identical, which proved that it was reasonable to apply the periodic boundary condition for expanding multi-scale analysis (material homogenization).

**Fig 9:**
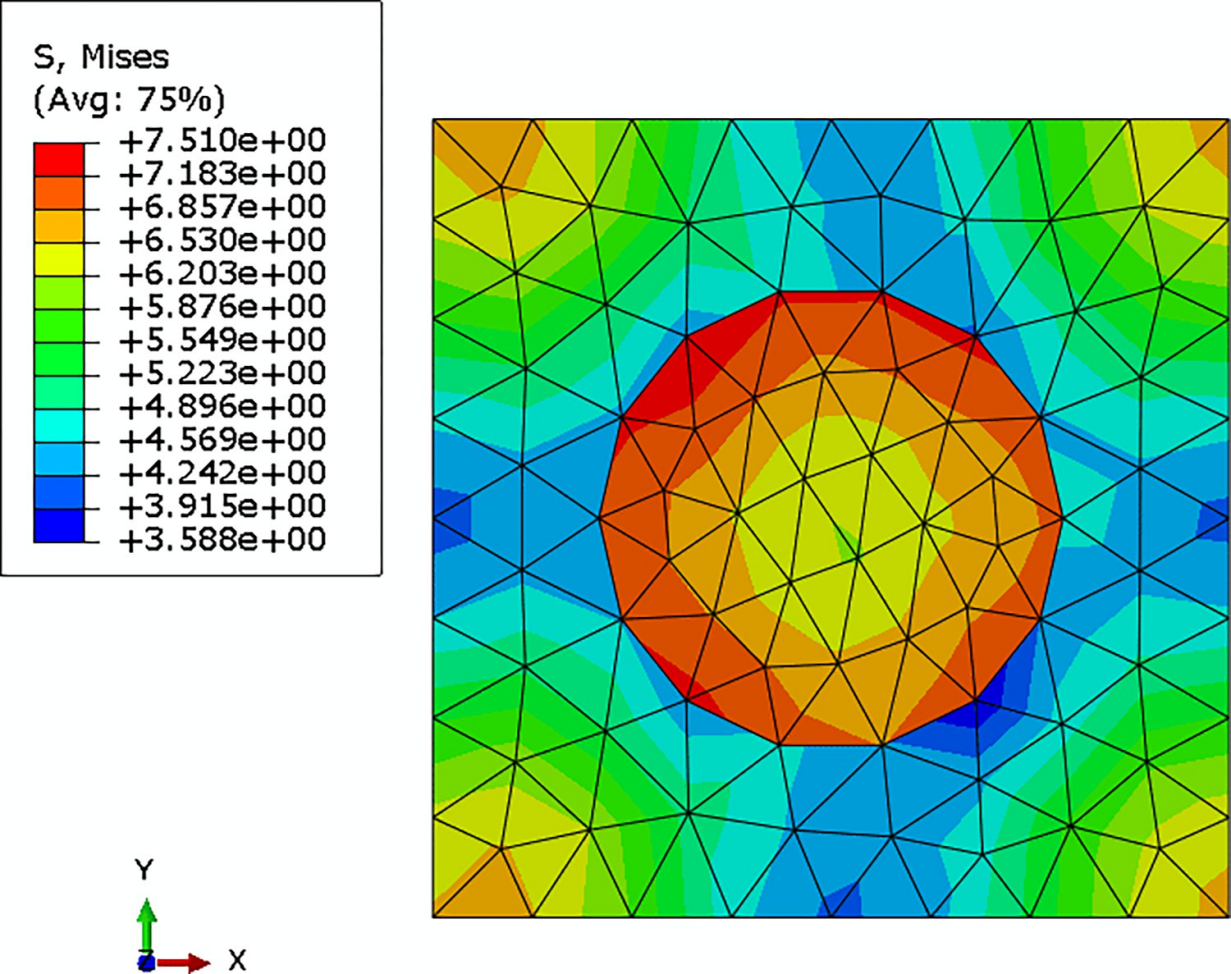
Local Mises stress field of the DNS model.

The mesoscopic stress field of the DNS model in the same region of the selected mesoscopic RVE was shown in Fig 9. Comparing Figs 7 with 9, it could be seen that the distribution of the mesoscopic stresses in the same region of the DNS model and the mesoscopic model were almost the same. The maximum relative difference between the two models was less than 2%. Since the computational cost of multi-scale models was much smaller than that of the DNS model, it was reasonable to adopt periodic boundary conditions for the contracting multi-scaling analysis.

### 3.3 Contracting multi-scale analysis of microcapsules in asphalt pavement

Based on the submodel method and the periodic boundary condition, a contracting multi-scale analysis from macro to meso was conducted. The stress field of the microcapsules in asphalt pavement was obtained, and the simplification method of the capsule core was validated by fluid-solid coupling in ABAQUS.

#### 3.3.1 Analysis of macro model

In order to obtain the strain components for the mesoscopic analysis, the analysis on the macro model was firstly conducted. It could be known from the above discussion that the incorporation of the microcapsules almost had no effect on the elastic constants of the asphalt mixture. Therefore, the modulus of the ordinary asphalt mixture could be directly regarded as the modulus of asphalt mixture with microcapsules. The material properties listed in Table 7 were adopted in this study.

**Table 7:** Parameters of pavement structure.

Pavement structure	Elastic modulus (MPa)	Poisson’s ratio	Thickness (cm)
Asphalt concrete surface (with 5%(mass percentage) capsules)	1820	0.311	12
Asphalt treated base	1200	0.300	18
Graded broken stone base	400	0.350	20
Lime-soil subbase	400	0.250	20
Subgrade	50	0.350	230

**Fig 10:**
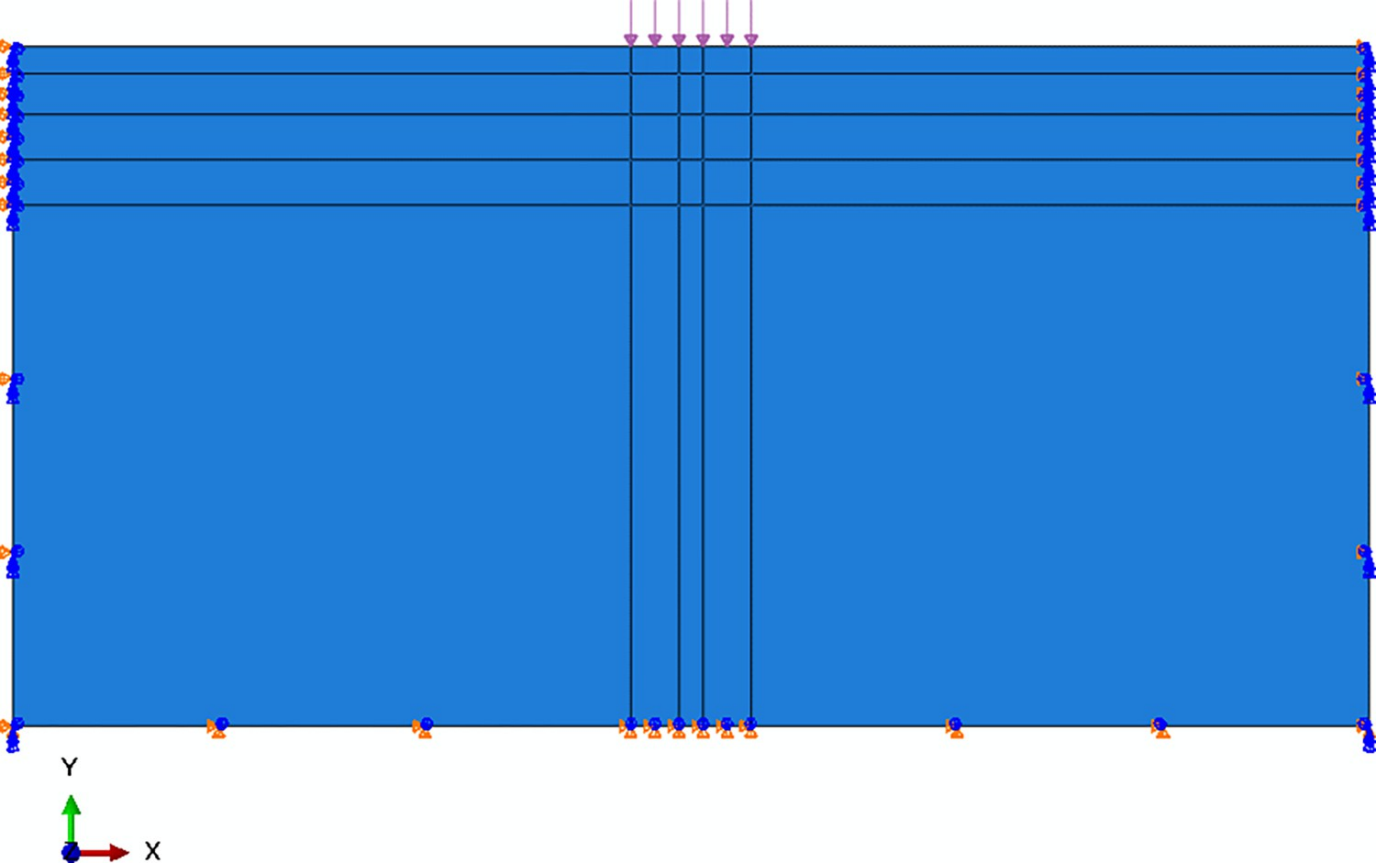
Macro FEM model of asphalt pavement.

Symmetrical and anti-symmetric boundary conditions were set on both sides and the bottom of the model, respectively. In addition, the vehicle load (double vertical loads with pressure of 700 kPa, radius of 0.1065 cm and gap of 0.1065 cm) was converted according to the principle of stress equivalence, and its intensity was determined as 117371 Pa/m. The geometry of macro model extended to 6 m and 3 m in the x and y direction. The model with the above boundary conditions was shown in Fig 10.

In this paper, the strain components of the fail element of the macro model were applied as the boundary condition of the mesoscopic model. There were several steps to obtain the required strain components in macro model. Firstly, a coarser mesh was adopted to obtain the stress field of the macro model. Then, referring to the mechanic of composite material [24] and the specification in China [28], the maximum tensile strain criterion was adopted to determine the stress sensitivity area in the macro model and the sub-model cutting boundary was furtherly obtained. Meanwhile, a finer mesh (mesh density of 4 mm was adopted in this study) was adopted in submodel, and its stress field was compared with that of the macro model to ensure the uniform transition of the stress field between macro model and the submodel at the cutting boundary. Finally, the strain components E11, E22, and E12 in the failure element of the submodel were obtained.

#### 3.3.2 Analysis of mesoscopic RVE

**Fig 11:**
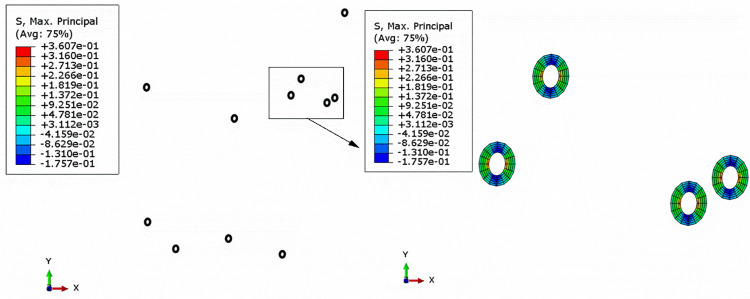
Maximum principal stress field of the capsule wall in mesoscopic model.

Based on the size of the mesh in submodel, the width and length of the mesoscopic model were determined as 4 mm. According to some reference [6–8,10,18,26,27] and the studies in section 3, the parameters of the capsule listed in Table 8 were adopted for the following analysis. Besides, the elastic constants of the matrix (asphalt mixture) were determined from Table 7. Then, according to Eqs (12)–(17), the strain components at the failure point were applied to the mesoscopic RVE. Assuming that the capsule wall was subjected to the maximum principal stress (MPS) criterion, the MPS field of the mesoscopic RVE were shown in Fig 11.

**Table 8:** Parameters of the microcapsule in mesoscopic model.

Parameter	Radius of whole capsule (mm)	Radius of capsule core (mm)	Modulus of capsule wall (GPa)	Poisson’s ratio of capsule wall	Modulus of capsule core (GPa)	Poisson’s ratio of capsule core
value	0.1	0.05	3.6	0.33	0.00135	0.4999

**Fig 12:**
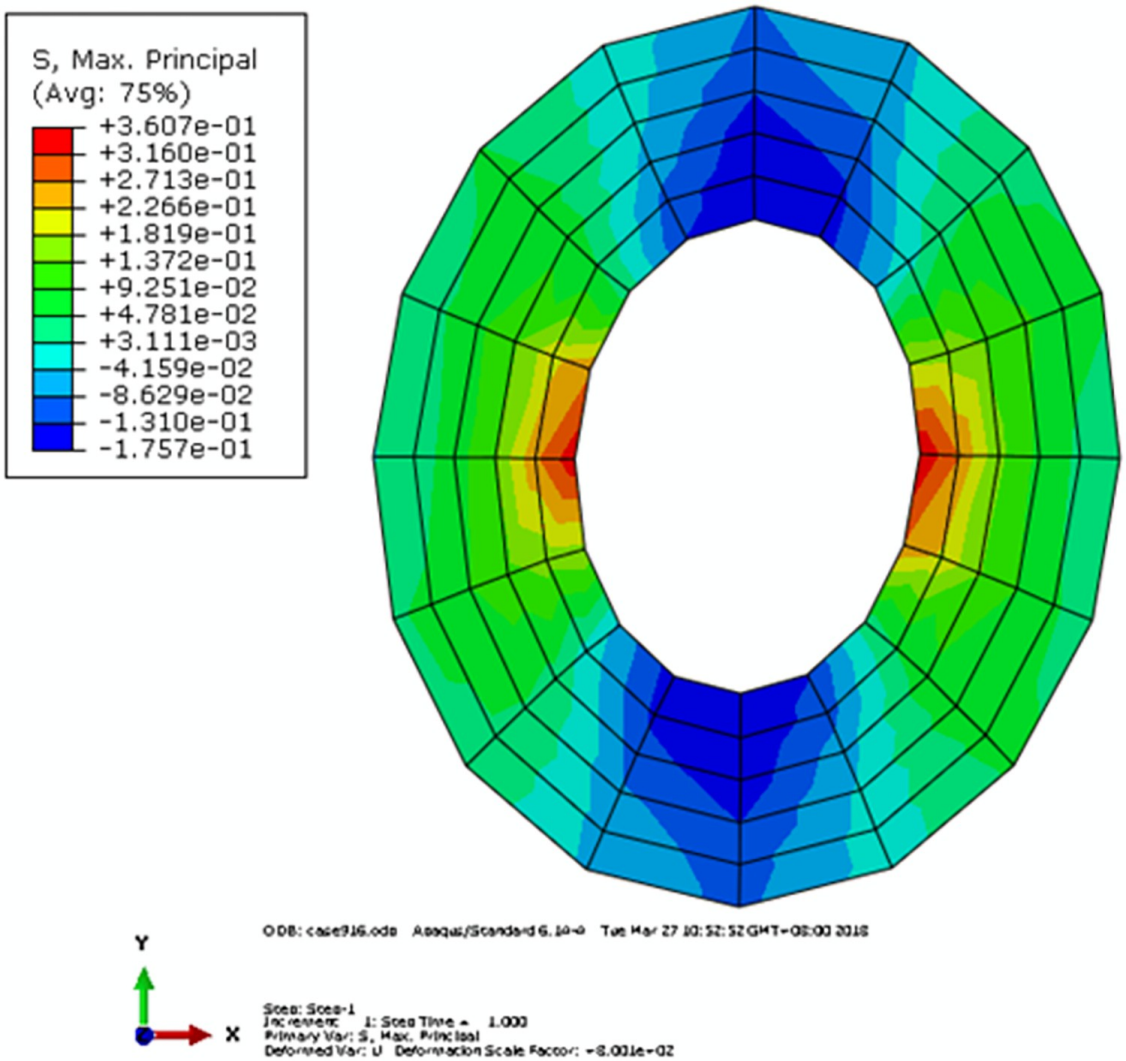
Maximum principal stress field of a single capsule in mesoscopic model.

It could be seen from Fig 11 that under the vehicle load, the maximum MPS of the capsule wall was 0.3607 MPa. In addition, the differences among the MPS of different capsules were not obvious, which indicated that there was no stress aliasing between different microcapsules. In order to further obtain the stress field of the capsule wall, the capsule with the maximum MPS on the capsule wall was selected for analysis, and its stress field was shown in Fig 12. It could be seen that there was an alternating area of tensile and compressive stress in the capsule wall and the absolute value of the stress on the inner side of the capsule wall was higher than that on the outer side. The MPS of the capsule wall was the tensile stress, which was symmetrically distributed and twice of the compressive stress. In addition, the tensile strength of the material was generally lower than its compressive strength. It could be concluded that when there was no crack in the road surface, the most vulnerable position of the capsule was located inside the capsule wall.

#### 3.3.3 Validation of the simplification method of capsule core

**Fig 13:**
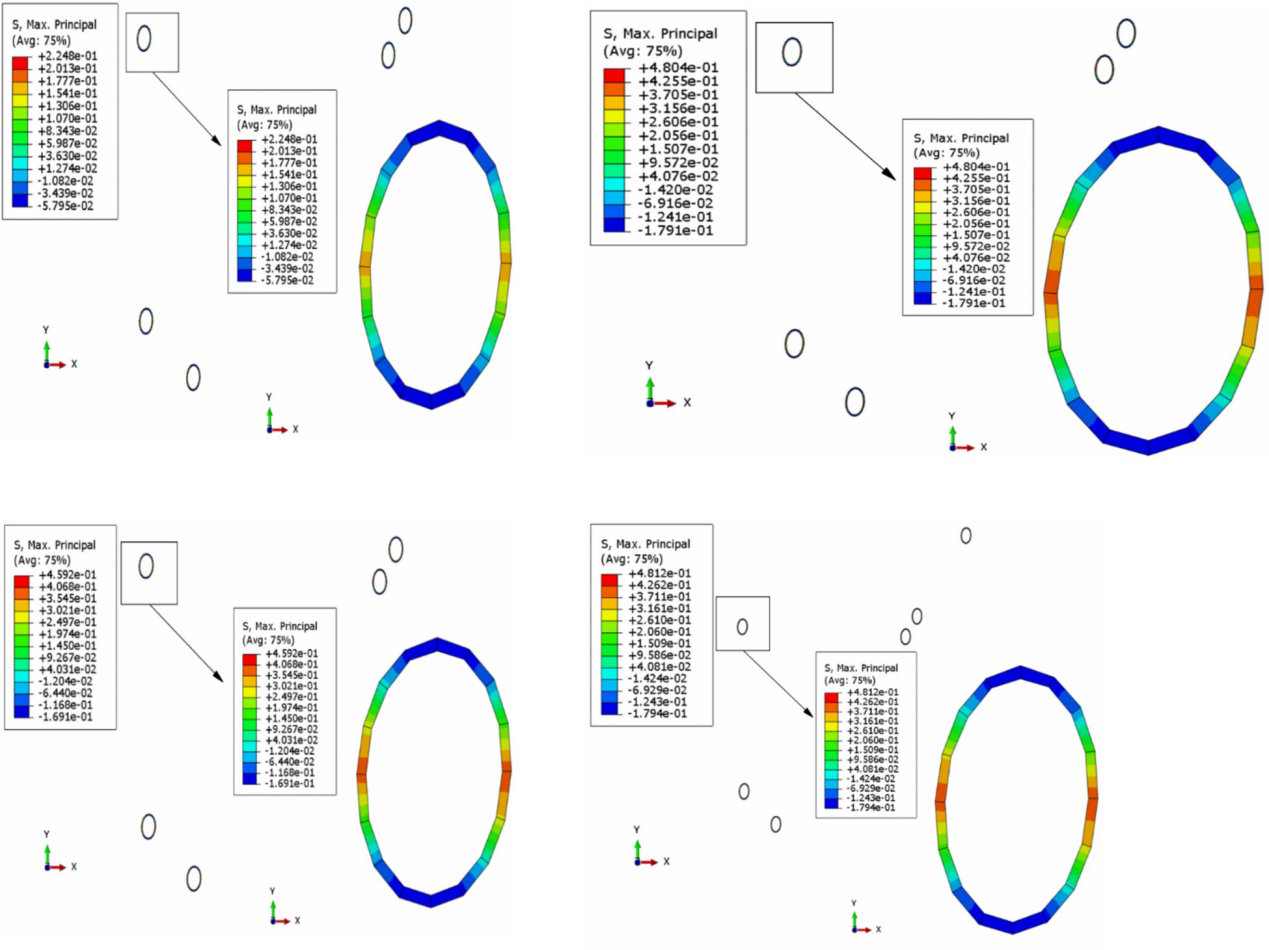
MPS of microcapsules of (*a*) Model A; (*b*) Model B; (*c*) Model C and (*d*) Model D.

If the capsule core was simplified to an approximately incompressible solid, its elastic constants could be determined by the method proposed in section 2. In the following study, the rationality of the determined elastic constants of the capsule core was further investigated according to the stress field of the capsule wall. The static fluid element F2D2 and the hybrid element CPE4RH were adopted to simulate the fluid and solid behavior of the capsule core, respectively. During the verification process, the volume ratio of capsule core was 0.729, and the diameter of the capsule was 0.15 mm, while the rest parameters of the capsule were the same with those adopted in mesoscopic RVE. Models named *A*, *B*, *C* and *D* were established, among which the capsule cores were simplified as void, approximately incompressible solid, incompressible solid [10], and liquid, respectively. Finally, the results of the MPS of the capsule wall were shown in Fig 13.

Fig 13 showed that the stress fields of model *B* and *D* were nearly the same. The results indicated that if the initial internal pressure of the microcapsule was ignored, the liquid core could be simplified as an approximately incompressible solid. Furthermore, the application of hybridization element during the simulation was easier than that of liquid element. Therefore, it was recommended to adopt an approximately incompressible solid with optimum elastic constants determined by the proposed model to simulate the capsule core.

### 3.4 Effects of the design parameters of the microcapsule on its crack resistance in asphalt pavement

In this section, the sensitivity analysis of the stress of capsule wall was conducted to determine the key design parameter which has greater effects on the stress filed of the capsule wall. Besides, according to some parameters in references, the crack resistance of microcapsules in asphalt pavement was validated.

#### 3.4.1 Sensitivity analysis of the stress of capsule wall

Three main design parameters for microcapsules, including the elastic modulus of the capsule wall, the relative radius of the capsule core and the capsule size, were selected in the sensitivity analysis of the maximum tensile stress of the capsule wall. The relative radius of the capsule core can be calculated from dividing the core radius by the whole capsule radius. Three levels were selected for each design parameter, and an orthogonal table was designed for analysis. All the combinations of parameters and results were listed in Table 9, where the number in parentheses represented the level of variables.

**Table 9:** Orthogonal table of the maximum tensile stress of the capsule wall.

Case	Capsule diameter (mm) (factor 1)	Relative radius of capsule core (factor 2)	Elastic Modulus of capsule wall (GPa) (factor 3)	Maximum tensile stress (MPa)
1	0.15(3)	0.7(2)	4(3)	0.458
2	0.15	0.5(1)	3.25(2)	0.340
3	0.15	0.9(3)	2.5(1)	0.403
4	0.1(2)	0.7	2.5	0.413
5	0.1	0.9	3.25	0.452
6	0.05(1)	0.5	2.5	0.301
7	0.05	0.7	3.25	0.429
8	0.05	0.9	4	0.529
9	0.1	0.5	4	0.407

Firstly, mean maximum tensile stresses of the capsule wall under a specific case was calculated and shown in Table 10. Comparing the mean maximum tensile stresses at different levels of each factor in Table 10, it could be seen that with the increase of the modulus of the capsule wall, the mean maximum tensile stress of the capsule wall increased from 0.372 MPa to 0.465 MPa. While with the decrease of the relative radius of the capsule core, the mean maximum tensile stress of the capsule wall increased from 0.349 MPa to 0.461 MPa. The change in the mean maximum tensile stress of the capsule wall caused by the change of capsule diameter was within 5%. Therefore, it can be seen that the capsule wall and the relative radius of the capsule core and the elastic modulus of the capsule wall were two key parameters in capsule design.

**Table 10:** Mean maximum tensile stresses of capsule wall under different cases.

Level	Capsule diameter (mm)	Relative radius of capsule core	Elastic modulus of capsule wall (MPa)
1	0.420	0.349	0.372
2	0.424	0.433	0.407
3	0.400	0.461	0.465

#### 3.4.2 Validation of the crack resistance of microcapsules in asphalt pavement

There are three main materials used for the wall of the self-healing microcapsule applied in asphalt pavement, which are urea formaldehyde resin, phenolic resin, and melamine formaldehyde resin [5–11]. The modulus of the above materials generally varies from 2.5 to 4 GPa and the tensile strength is generally greater than 10 MPa [10,24]. When the wall material is selected, the relative diameter of the capsule can be changed by changing the mixing rate, potential of hydrogen (PH), and the ratio of the raw material. Eventually, the maximum tensile stress of the capsule wall can be changed and the crack resistance of the capsule wall can be controlled. However, it could be known from the calculation that when there were no microcracks in asphalt pavement, the maximum tensile stress of the capsule wall was generally less than 1 MPa under vehicle load. The value was much smaller than the tensile strength of the capsule wall, which indicated that in asphalt pavement, the microcapsules with resin as the capsule wall would not break on the early stage.

## 4. Conclusions

Multi-scale methods, including expanding the multi-scale method and contracting multi-scale method, were proposed in this study to investigate the crack resistance of the microcapsule in asphalt pavement. The main conclusions were listed as follows:

1. The elastic modulus of the capsule wall and the volume ratio of the capsule core had a great influence on the equivalent elastic constants of the microcapsule. During the numerical analysis, the core could be simplified to the approximately incompressible solid, and when the bulk modulus of the core was 2.25 GPa, its elastic modulus and Poisson’s ratio were 1.35 MPa and 0.4999 respectively. The effect of microcapsules on the elastic constant of the asphalt mixture was negligible.
2. According to the macroscopic displacement and mesoscopic stress field comparison of the established two-scale and full-scale orthotropic SiC/Ti cantilever beam model, the periodic boundary conditions could be used in expanding and contracting multi-scale analysis.
3. There was no stress aliasing between the microcapsules in the asphalt pavement, and the maximum tensile stress of the capsule wall was symmetrically distributed inside the capsule wall. With the increase of the modulus of the capsule wall, the mean maximum tensile stress of the capsule wall increased from 0.372 MPa to 0.465 MPa, while with the decrease of the relative radius of the capsule core, the mean maximum tensile stress of the capsule wall increased from 0.349 MPa to 0.461 MPa. The change in the mean maximum tensile stress of the capsule wall caused by the change of capsule diameter was within 5%. The relative radius of the capsule core and the elastic modulus of the capsule wall were two key parameters in capsule design.
4. On the initial stage of the asphalt pavement, the maximum value of tensile stress of the resin capsule wall was less than 1 MPa, while the tensile strength of the capsule wall was generally higher than 10 MPa. Therefore, the resin microcapsule in asphalt pavement would not break under the load of the vehicle before it encountered microcracks.
